# Case report: Response to endocrine therapy in triple-negative breast cancer metastases with altered hormone receptors

**DOI:** 10.3389/fonc.2023.1023787

**Published:** 2023-02-14

**Authors:** Ruoyan Qin, Jie Qian, Mengjun Shan, Guangxin Ruan, Xiaofeng Yang, Yanwen Wang, Lingshuang Liu

**Affiliations:** ^1^ Department of Oncology, Longhua Hospital, Shanghai University of Traditional Chinese Medicine, Shanghai, China; ^2^ Department of Emergency Medicine, Shanghai Chest Hospital, Shanghai Jiao Tong University School of Medicine, Shanghai, China; ^3^ Department of Pathology, Longhua Hospital, Shanghai University of Traditional Chinese Medicine, Shanghai, China

**Keywords:** triple-negative breast cancer, metastases, altered hormone receptors, later-line treatment, endocrine therapy

## Abstract

Triple-negative breast cancer refers to breast cancer patients with negative estrogen receptor (ER), progesterone receptor (PR) and human epidermal growth factor receptor (HER2). Metastatic triple-negative breast cancer is predominantly treated with chemotherapy, but later-line treatment remains challenging. Breast cancer is highly heterogeneous, and the expression of hormone receptors is often inconsistent between primary and metastatic lesions. Here, we report a case of triple-negative breast cancer 17 years after surgery with lung metastases for 5 years that progressed to pleural metastases after multiple lines of chemotherapy. The pleural pathology suggested ER (+) and PR (+) and transformation to luminal A breast cancer. This patient received fifth-line letrozole endocrine therapy and achieved partial response (PR). The patient’s cough and chest tightness improved after treatment, associated tumor markers decreased, and progression-free survival (PFS) exceeded 10 months. Our results may be of clinical relevance for patients with hormone receptor alterations in advanced triple-negative breast cancer and suggest that individualized regimens should be developed for breast cancer based on the molecular expression of tumor tissue at the primary and metastatic sites.

## Highlights

- Triple-negative breast cancer may exhibit postoperative metastatic hormone receptor alteration
- Individualized treatment based on re-evaluated primary tumor and metastatic hormone receptors may have survival benefit for patients with triple-negative breast cancer

## Introduction

Breast cancer is the most common malignancy in women in terms of incidence and has the second highest mortality (1). The treatment strategy is based on molecular typing, with the expression of estrogen receptor (ER), progesterone receptor (PR) and human epidermal growth factor receptor 2 (HER2) being the most important. These receptors define four different subtypes of breast cancer: luminal A (ER/PR positive, Her2 negative), luminal B (ER and/or PR positive, HER2 positive), HER2 overexpressing (HER2 positive only) and triple negative (2), each with a different treatment strategy and prognosis. Triple-negative breast cancer accounts for 15%-20% of all breast cancers (3) and is characterized by a higher risk of recurrence and poorer prognosis (4). Later-line treatment options are limited and poorly tolerated. In the treatment of advanced breast cancer, biological marker inconsistencies between the primary and metastatic breast cancer sites are often identified, which may impact the treatment strategy and prognosis of metastatic breast cancer, leading to altered treatment outcomes and results. Loss of hormone receptors may lead to a poorer prognosis. Patients with positive ER and PR transitions may benefit from endocrine therapy, and HER2-positive patients may choose to receive targeted therapy.

Here, we present a patient with triple-negative breast cancer who exhibited postoperative metastatic hormone receptor changes. The patient benefited from endocrine therapy, suggesting that a re-biopsy should be performed for recurrent metastatic lesions to reassess the molecular status (5).

## Case presentation

**Figure 1:**
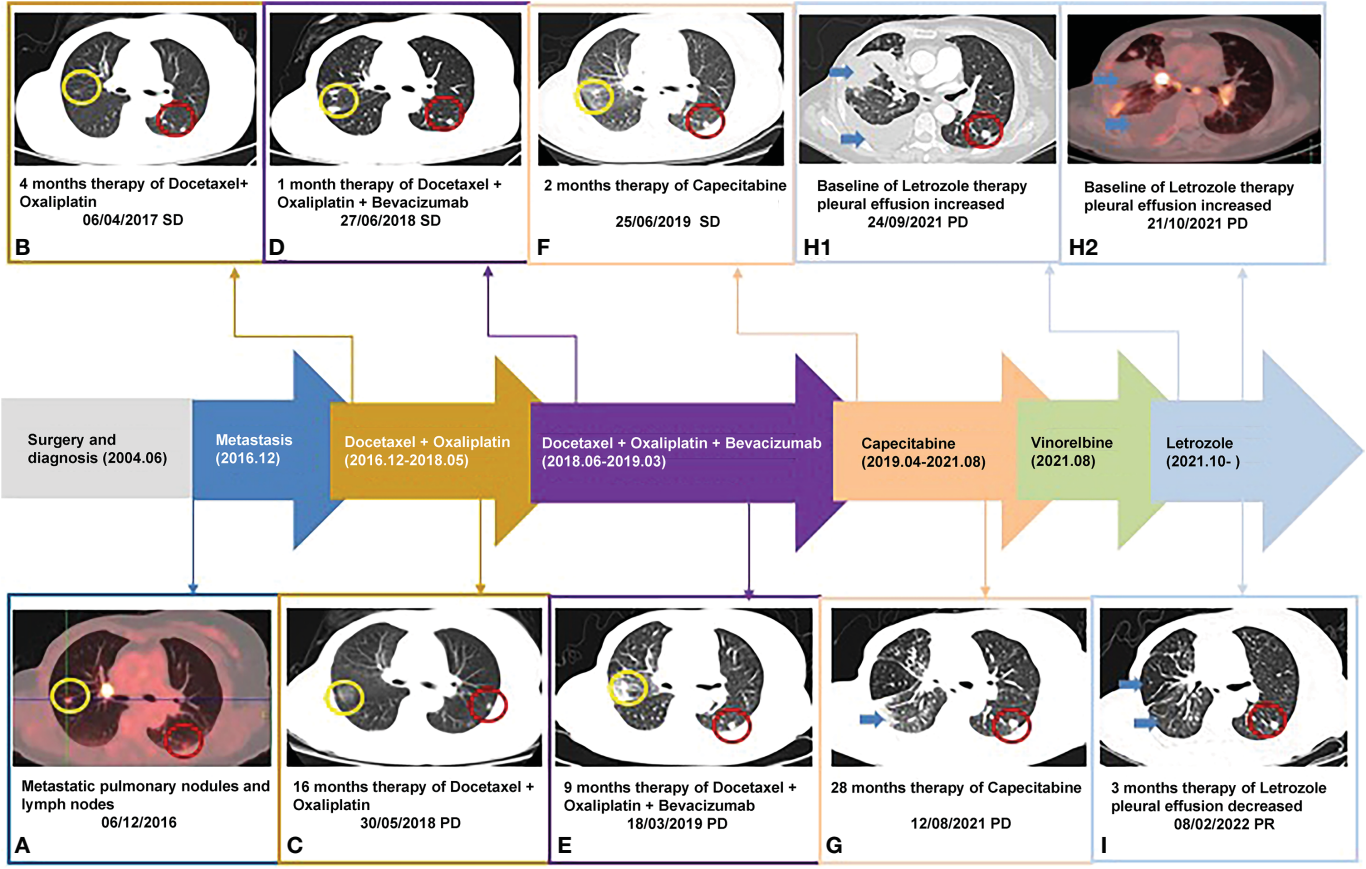
Timeline of the patient: **(B-G, H1, I)** were taken with Chest CT scanner and **(A, H2)** were taken with PET-CT scanner; yellow circle: right pulmonary nodule; red circle: right pulmonary nodule; blue arrow: pleural effusion.

A 67-year-old Chinese woman was admitted to Longhua Hospital of Shanghai University of Traditional Chinese Medicine on September 27, 2021, complaining of cough and chest tightness for 1 week. The patient had a history of postoperative right breast cancer of 17 years with metastasis in both lungs for more than 5 years. She underwent right breast lump resection and modified radical right mastectomy on June 4, 2004. The resected lump was 3*3*2.8 cm^3^ in size, and pathology showed grade III invasive ductal carcinoma (right breast). Fifteen ipsilateral axillary lymph nodes were identified during the operation and showed no sign of malignancy on pathological examination. Immunohistochemistry revealed ER (-), PR (-), SMA(-),EMA(+), S-100(-),P53(-),C-erbB-2(-),bcl-2(-),nm23(+),Ki-67(-),AE1/AE3(+),Vimentin(+), GFAP (-). At that point, the stage was p-T2N0M0 IIA. The patient was treated with a CMF regimen (CTX600mg+MTX30mg+5-Fu500mg ivgtt q3w). Adjuvant chemotherapy was given 6 times after surgery, followed by a regular follow-up review. The patient underwent PET-CT (Positron Emission Computed Tomography) on December 6, 2016, showing mild FDG metabolism in small nodules in the posterior segment of the upper right lung lobe, possibly indicating malignancy (1.1 cm, SUVmax1.9). Multiple small nodules were also identified in the basal segment of the lower right lobe and in the subpleural area of the lower left lobe and upper left lobe (7mm, SUVmax2.2). In addition, multiple enlarged lymph nodes with increased FDG metabolism were observed in the mediastinum and right hilar region (1.6*1.3 cm, SUVmax19.5) (**Figure 1A**). The patient refused to undergo diagnostic puncture and was re-staged r-T0N0M1 stage IV (lung, mediastinum, hilar lymph nodes) according to imaging. She received first-line DO chemotherapy (docetaxel 60mg d1d8+oxaliplatin 100mg d1d8 ivgtt q3w), achieving stable disease (SD). The first-line treatment resulted in 16 months of progression-free survival (PFS) (**Figures 1B, C**). The patient’s second-line treatment was based on the first-line treatment plus bevacizumab (docetaxel 60mg d1d8 + oxaliplatin 100mg d1d8 + bevacizumab 300mg d1 ivgtt q3w), which also achieved SD and led to 9 months of PFS (**Figures 1D, E**). The third-line therapy was capecitabine (capecitabine 1.5g BID PO d1-d14 q3w), which also achieved SD. In April 2021, computed tomography (CT) scans revealed increased exudate in the lower lobe of the right lung, and new right pleural effusion, indicating progressive disease (PD). Following the third-line treatment, the patient demonstrated a PFS of 28 months (**Figures 1F, G**). She was then treated with fourth-line vincristine soft gels (vincristine 100mg qw PO q3w). On the night of the same day, she developed increased hoarseness, fever, nausea, and diarrhea 7-8 times. Therefore, vincristine was stopped, and her condition improved with symptomatic treatment.

**Figure 2:**
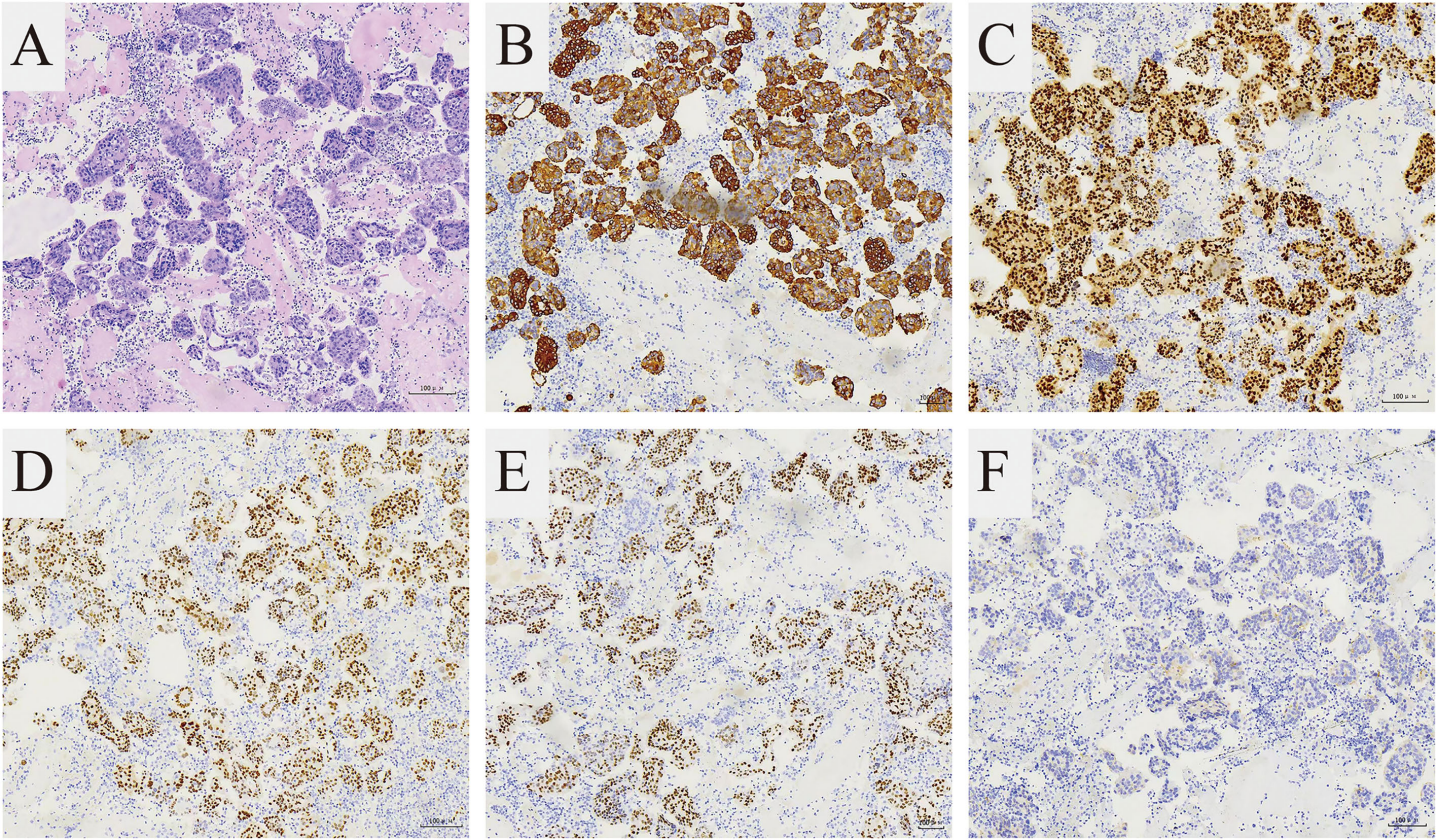
Pathological features of the cell blocks of the pleural fluid. **(A)** The H and E stain showed that the tumor is composed of small round cells (magnification 100×). The immunohistochemical stain showed **(B)** CK7 (+) and **(C)** GATA3 (+) supporting breast cancer metastasis (magnification 100×). The immunohistochemical stain showed **(D)** ER (+), **(E)** PR (+), **(F)** HER-2(1+) representing Luminal A subtype (magnification 100×).

**Figure 3:**
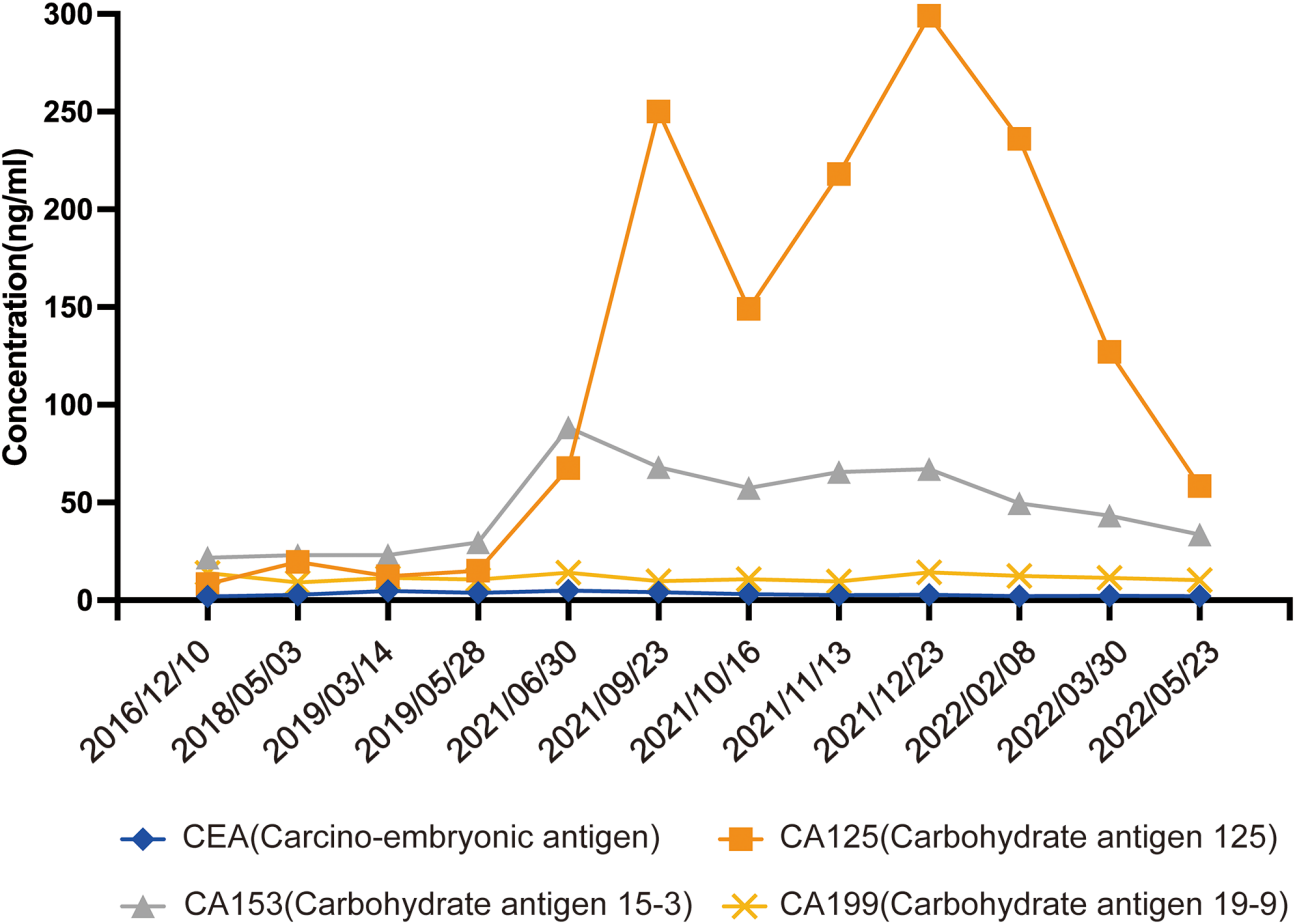
Serum tumor biomarker during treatment.

In September 2021, the patient attended the hospital due to aggravation of coughing and chest tightness for 1 week and underwent right-sided thoracentesis and drainage. Physical examination revealed low breathing sound in right lung. The patient received symptomatic support treatment including cough relieving and considerate nursing. 510 ml of bloody pleural fluid was drained, and the pathology of pleural fluid (pleural cell mass) suggested metastatic adenocarcinoma. Combined with the immunostaining results, cell morphology and clinical history, the findings were consistent with breast cancer metastasis. Immunohistochemistry showed ER(+,90% strong positive), PR(+,80% strong positive), HER-2.(1+), Ki-67(10%+), CK20(-), GATA3(+), SOX10(-), CK7(+), TTF-1(-), EMA(+), calretinin(mesothelial+), WT-1(mesothelial+) (**Figures 2A–F**). PET-CT revealed a soft tissue mass in the right hilar lung (26.7*38.4mm, SUVmax 20.05), focal high-density nodules in the right anterior chest wall (14*8mm, SUVmax 3.67), multiple nodules in both lungs, multiple lymph nodes in the bilateral clavicular region, bilateral hila and mediastinum (12mm, SUVmax17.06), and multiple lesions in the right pleura with abnormally high FDG metabolism (SUVmax 13.52) which were considered to be caused by multiple tumor metastases (**Figures 1H1, H2**). The patient was started on fifth-line letrozole endocrine therapy since October 2021. In February 2022, CT scans revealed decreasing right upper lung hilar soft tissue shadow, right pleural fluid and mediastinal right hilar lymph nodes (**Figure 1I**). Treatment efficacy was categorized as partial response (PR). The patient’s cough and chest tightness were significantly relieved. Physical examination showed clear breath sounds in both lungs. The related tumor markers CA153 and CA125 showed a decreasing trend (**Figure 3**). Currently, the PFS reached 10 months, and the patient is still under follow-up.

## Discussion

Triple-negative breast cancer is characterized by a lack of estrogen, progesterone and HER2 receptor expression, resulting in ineffective endocrine and HER2-targeted therapies. Chemotherapy remains the most common basic treatment for triple-negative breast cancer, including single agent and combination chemotherapy based on paclitaxel, capecitabine, and vincristine, which can be combined with bevacizumab anti-angiogenesis. However, drug resistance inevitably occurs, with limited later-line treatment options and poor survival prognosis. During the course of breast cancer, hormone receptor expression in metastases may differ from the primary lesion. Given this inconsistency, the ASCO expert group, Chinese Consensus Guidelines for Advanced Breast Cancer, recommends re-biopsy of metastases to determine treatment based on the ER and PR status of the metastases. However, there are still many barriers to biopsy of recurrent tumor metastases (6), such as the risk of major complications, patient refusal, differences in puncture detection techniques, and low representativeness of tissue specimens. In this case, the patient experienced a 17-year tumor course with postoperative bilateral lung metastases from triple-negative breast cancer. Following multiple lines of chemotherapy resistance and intolerance, the tumor metastasized to the pleura, with pleural fluid pathology immunohistochemistry revealing CK7 (+) and GATA3 (+), supporting breast cancer metastasis. However, the results ER (+) and PR (+) supported a transformation to luminal A breast cancer, which was treated according to hormone receptor alterations to benefit from letrozole endocrine therapy.

The inherent heterogeneity of breast cancer and the changes that occur during its evolution result in distinct biological features between primary and metastatic foci. The hormone receptors ER and PR are important prognostic factors and predictors of endocrine therapy efficacy in breast cancer, suggesting the importance of detecting the expression of hormone receptors. We analyzed 15 relevant studies from 2010 to 2020 and showed that the receptor expression in recurrent metastatic breast cancer lesions varied to different degrees compared to the primary lesions (**Table 1**) (7–21). Relatively high inconsistency was observed for hormone receptors, especially PR, and relatively low inconsistency for HER2; hormone receptor loss was more common than hormone receptors turning positive. Yeung reviewed (22) 47 studies of paired primary and metastatic sites (3384 cases) and came to similar conclusions: the rates of inconsistency were 14%, 21%, and 10% for ER, PR, and HER2, respectively, and the rates of decrease and increase in receptor expression were 9.17% and 4.51%, respectively. The results also demonstrated that the rates of receptor inconsistency differed between metastatic sites, suggesting that inconsistency is a real biological phenomenon. The inconsistency of hormone receptors may affect the treatment and management of patients, highlighting the possibility of potential therapeutic agents (17). Consistent with previous literature, it is rare for this patient to change from triple negative breast cancer to hormone receptor positive.

**Table 1:** Literature review of ER, PR and HER2 discordance between primary tumors and corresponding metastatic sites.

Reference	Cases	Metastatic sites	Type of analysis	ER (Gain/Loss) (%)	PR (Gain/Loss) (%)	HER2 (Gain/Loss) (%)	Change in Therapy (%)
Thompson 2010 (7)	137	LR/DM	Biomarkers reassessment	10.2 (2.2/8.0)	24.8 (8.8/16.0)	2.9 (2.2/0.7)	17.5
Amir 2011 (8)	231	LR/DM	Reports review	12.6 (3.0/9.6)	31.2 (7.0/24.2)	5.5 (4.1/1.4)	14.2
Amir 2011 (9)	94	LR/DM	Biomarkers reassessment	16.0 (4.3/11.7)	40.4 (4.2/36.2)	9.6 (7.2/2.4)	14
Bogina 2011 (10)	140	LR/DM	Biomarkers reassessment	6.4 (0.7/5.7)	21.4 (3.6/17.8)	0.7 (0.7/0)	7.3
Dieci 2012 (11)	119	LR/DM	Biomarkers reassessment	13.4 (2.5/10.9)	39.0 (8.5/30.5)	11.8 (8.4/3.4)	10.9
Hoefnagel 2012 (12)	233	DM	Biomarkers reassessment	10.3 (-/-)	30.0 (-/-)	-(-/-)	–
Curtit 2013 (13)	235	LR/DM	Biomarkers reassessment	17.0 (4.7/12.3)	29.3 (7.2/22.1)	4 (1.0/3.0)	–
Duenas 2014 (14)	184	LR/DM	Biomarkers reassessment	21.3 (12.5/8.8)	34.6 (12.8/21.8)	16.4 (10.0/6.4)	31
Shiino 2016 (15)	153	LR/DM	Biomarkers reassessment	18.3 (3.9/14.4)	26.1 (6.5/19.6)	6.5 (3.9/2.6)	–
Erdem 2016 (16)	393	LR/DM	Biomarkers reassessment	27.2 (12.2/15.0)	38.6 (10.2/28.4)	14.4 (10.1/4.3)	–
McAnena 2018 (17)	132	LR/DM	Biomarkers reassessment	20.4 (4.5/15.9)	37.7 (4.5/33.2)	3 (1.5/1.5)	6.8
Woo 2019 (18)	152	LR/DM	Biomarkers reassessment	6 (0.7/5.3)	26.3 (2.0/24.3)	7.9 (2.0/5.9)	–
Nguyen 2019 (19)	67	LR/DM	Biomarkers reassessment	26.9 (14.9/12.0)	38.8 (13.4/25.4)	22.4 (14.9/7.5)	–
Jud 2020 (20)	142	LR	Biomarkers reassessment	14.9 (-/-)	22.7 (-/-)	18.3 (-/-)	–
Blancas 2020 (21)	45	LR/DM	Biomarkers reassessment	20 (8.9/11.1)	20 (4.4/15.6)	15.4 (5.1/10.3)	–

LR, local recurrences; DM, distant metastases; ER, estrogen receptor; PR, progesterone receptor.

The variation in metastatic receptor expression may result from several factors, including differences in sampling and detection, tumor heterogeneity, and antitumor therapy. In terms of sampling and detection, differences in sampling methods of tissue specimens, representativeness of sampling, immunohistochemical staining, and accuracy of detection methods are inevitable. Tumor heterogeneity refers to changes in tumors during continuous proliferation and differentiation and changes in molecular biological characteristics or genetic level. Tumor heterogeneity can be divided into spatial heterogeneity (different regions of the same tumor) and temporal heterogeneity (discrepancy between primary tumors and secondary tumors). The underlying mechanisms are mainly believed to involve the clonal evolutionary theory and the stem cell theory, in which the tumor microenvironment and the tumor treatment process play major roles. The above theories explain the inconsistency between the receptors of metastatic and primary foci of breast cancer. Curtit (13) reported that previous chemotherapy, especially anthracycline-based chemotherapy, was significantly associated with alterations in ER receptors. Our patient presented with a long postoperative metastatic course and had received CMF adjuvant chemotherapy, docetaxel-based chemotherapy and capecitabine chemotherapy. Therefore, the changes in metastatic receptors might be associated with tumor heterogeneity and chemotherapy history.

Previous studies have reported the impact of inconsistent receptor expression on subsequent treatment and prognosis. Considering that the presence of ER, PR or HER2 expression in metastases suggests an opportunity for patients to receive endocrine therapy or targeted therapy, treatment resistance may occur when receptor status is lost. In 6.8-31% of patients, the treatment strategy is switched due to changes in molecular markers (7–11, 14, 17). In terms of prognosis, Bogina (10) showed that among patients with local recurrence and primary ER, patients whose recurrence foci turned negative for PR had a significantly shorter median distant metastasis-free survival (MFS) than those who remained PR positive (p= 0.005). Dieci (11) showed that patients with hormone receptor ER/PR and HER2 loss had shorter overall survival (OS) (P = 0.06 and P = 0.0002) and post-recurrence survival (PRS) after relapse (P=0.01 and P=0.008) than those without hormone receptor loss. McAnena (17) also came to similar conclusions, reporting that luminal A breast cancer patients who converted to triple-negative breast cancer had significantly worse survival after recurrence than those with persistent luminal A breast cancer (P < 0.05). Furthermore, the difference in overall survival was close to statistical significance (P=0.064). Hoefnagel (12) showed that patients who changed ER/PR receptor status to negative or to positive had a similar prognosis to patients with persistent negative receptor expression but shorter overall survival compared to patients with persistently positive receptor expression. Positive primary hormone receptors ER and PR indicate a good prognosis, while negative status indicates a poor prognosis. Loss of receptors in recurrent metastases also appears to be associated with poor prognosis. There are fewer data related to the prognostic impact of receptor acquisition.

In conclusion, this case report highlights a partial response to endocrine therapy in a patient with triple-negative breast cancer metastases with altered hormone receptors.

Our research suggests that re-evaluation of the diagnosis based on primary tumor and metastatic hormone receptors is the key to implementing individualized treatment of tumor heterogeneity, especially for triple-negative breast cancer. This subject is worthy of further clinical research and discussion.

## Data availability statement

The raw data supporting the conclusions of this article will be made available by the authors, without undue reservation.

## Ethics statement

Ethical review and approval was not required for the study on human participants in accordance with the local legislation and institutional requirements. The patients/participants provided their written informed consent to participate in this study.

## Author contributions

RQ, JQ and LL: Conceptualization, methodology, and review. RQ, XY, and YW: Data collection and analysis, writing, and editing. GR and MS: Literature research. All authors contributed to the article and approved the submitted version.
